# Source of Variant Creutzfeldt-Jakob Disease outside United Kingdom

**DOI:** 10.3201/eid1308.070178

**Published:** 2007-08

**Authors:** Pascual Sanchez-Juan, Simon N. Cousens, Robert G. Will, Cornelia M. van Duijn

**Affiliations:** * Erasmus Medical Center, Rotterdam, the Netherlands; † University Hospital “Marqués de Valdecilla,” Santander, Spain; ‡ London School of Hygiene and Tropical Medicine, London, UK; § Western General Hospital, Edinburgh, UK

**Keywords:** variant Creutzfeldt-Jakob disease, bovine spongiform encephalopathy, live bovine imports, mechanically recovered meat, research

## Abstract

We studied the occurrence of variant Creutzfeldt-Jakob disease (vCJD) outside the United Kingdom in relation to the incidence of indigenous bovine spongiform encephalopathy (BSE) and to the level of live bovines and bovine products imported from the UK during the 1980s and the first half of the 1990s. Our study provides evidence that a country’s number of vCJD cases correlates with the number of live bovines it imported from the UK from 1980 to 1990 (Spearman rank correlation coefficient [r_s_] 0.73, 95% confidence interval [CI] 0.42–0.89, p<0.001). Similar correlations were observed with the number of indigenous BSE cases (r_s_ 0.70, 95% CI 0.37–0.87, p = 0.001) and carcass meat imported from the UK from 1980 to 1996 (r_s_ 0.75, 95% CI 0.45–0.89; p<0.001) Bovine imports from the UK may have been an important source of human exposure to BSE and may have contributed to the global risk for disease.

## Abstract 2

Bovine imports during the 1980s and the first half of the 1990s from the UK contributed substantially to the global spread of this disease.

In 1996 a new variant of Creutzfeldt-Jakob disease (vCJD) was described in the United Kingdom (*1*). By September 2006, 196 cases had been reported worldwide; most (162, 83%) occurred in the UK. Laboratory and epidemiologic studies provide strong circumstantial evidence for a causal link between vCJD and the bovine spongiform encephalopathy (BSE) epizootic in cattle (*2*,*3*) with the most likely route of primary human infection being through dietary exposure to highly infected bovine tissues (*3*).

In recent years, vCJD has been identified in a number of European countries with indigenous outbreaks of BSE, including 20 cases of vCJD in France, 4 in Ireland, 2 in the Netherlands, and single cases in Portugal, Italy, and Spain. A growing number of cases of vCJD have also been identified in countries outside Europe that have minimal incidence of BSE, including Japan, the United States, and Canada, and also in Saudi Arabia, a country in which BSE has not been identified (*4*). Several of the non-UK patients, notably 2 Irish, 1 Canadian, 2 American, and possibly the Japanese patient, may have been infected during periods of residence in the UK, but most cases (28 of 34) occurring outside the UK were in persons who had never visited the UK. Although vCJD cases have occurred in countries with very low incidence of BSE, no cases have been reported in countries with higher incidence of BSE, such as Switzerland (460 reported cases of BSE) and Germany (395 cases) (*5*). This fact raises questions as to the source of infection in the vCJD cases outside the UK. Were the patients exposed to indigenous cases of BSE or to infected bovine material imported from the UK? An analysis of infection risk in France suggests that the most likely source of vCJD in that country is imported infected material from the United Kingdom (*6*). In the Republic of Ireland, the transmission of BSE to humans was estimated to be equally likely from indigenous BSE or from UK imports (*7*). Given the apparently weak association between the occurrence of indigenous BSE and vCJD in some countries, we studied the occurrence of vCJD cases outside the UK in relation to the level of imported bovines and bovine products from the UK during the 1980s and the first half of the 1990s.

## Methods

A European Union (EU) surveillance network, established in 1993, ensures prospective surveillance for CJD by using standard methods (*8*) in 18 European countries and the United States, Canada, Israel, and Australia. By August 24, 2006, 32 cases of vCJD had been identified in these countries (excluding the UK) (Table 1). Two more cases had been identified in countries outside the surveillance network, Japan and Saudi Arabia. Case-patients in Canada (n = 1), the United States (n = 2), Ireland (n = 2), and possibly Japan (n = 1) were considered as likely to have been infected during periods of residence in the UK.

**Table 1:** Worldwide variant Creutzfeldt-Jakob disease cases as of August 2006

Country of residence at disease onset	N
United Kingdom	162
France	20
Republic of Ireland	2+2*
Italy	1
United States	2*
Canada	1*
Saudi Arabia	1
Japan	1*
The Netherlands	2
Portugal	1
Spain	1

*Most likely exposed to bovine spongiform encephalopathy in the UK.

Data on indigenous BSE cases detected by both passive and active surveillance were obtained from the World Organization for Animal Health webpage (www.oie.int/eng/info/en_esbmonde.htm) (*5*). The number of live cattle and the tonnage of carcass meat exported from the UK were derived from UK Custom and Excise Data (*9*).

We included in our analysis all countries covered by the EU surveillance network. Because exposure was expressed as a total amount rather than per capita, we based the analysis on number of vCJD cases rather than rates. We plotted the incidence of vCJD (number of cases) by country against the following factors: 1) number of cases of indigenous BSE, 2) number of live bovines imported from the United Kingdom from 1980 to 1990, and 3) tonnage of carcass meat imported from the United Kingdom from 1980 to 1996. For the last 2 plots, we used logarithmic scales to improve visualization of the data.

We have included carcass meat data until 1996, when all UK bovine imports were banned by the EU. However, for live bovine imports we limited our analysis to the period 1980–1990. In 1990 the EU restricted live bovine exports from the UK to animals <6 months of age and required that the importing country must ensure that any imported cattle from the UK were slaughtered at <6 months of age (*10*). For the correlation analyses, we included only the non-UK vCJD patients who are thought likely to have acquired infection outside the UK. A factor likely to be important for BSE exposure is the temporal distribution of UK exports. The number of BSE-infected cattle entering the human food supply is estimated to have peaked in the UK around 1989 (*7*), although with respect to exports, peak exposure may have been later, around 1992–1993. For example, 85% of Germany’s imports of UK carcass meat between 1980 and 1990 were before 1988. In contrast, 70% of the livestock imports to the Netherlands were from 1987 through 1990. To account for this fact, we weighted the number of live bovines and the carcass meat tonnage imported each year by the size of the UK BSE epizootic that year (reported clinical cases) and normalized the data by the maximum number of BSE cases detected in a year during the epizootic. Nonparametric Spearman rank correlation coefficients (r_s_) were calculated to evaluate whether evidence of a correlation between exposure and outcome existed.

## Results

**Figure 1:**
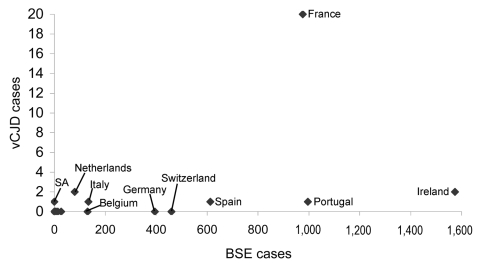
Scatter plot of the number of cases of indigenous bovine spongiform encephalopathy (BSE) in non–UK countries and the number of non-UK variant Creutzfeldt-Jakob disease (vCJD) cases per country. SA, Saudi Arabia.

**Figure 2:**
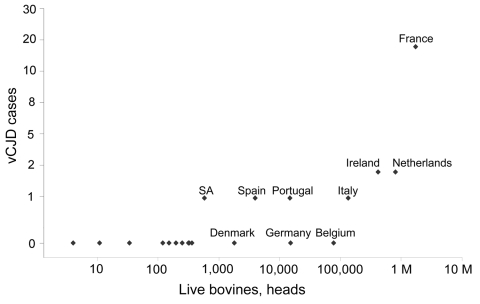
Scatter plot of live bovine imports (unweighted data) from the UK (1980–1990) and the number of non-UK variant Creutzfeldt-Jakob disease (vCJD) cases per country. Values are logarithmic. M, million.

**Figure 3:**
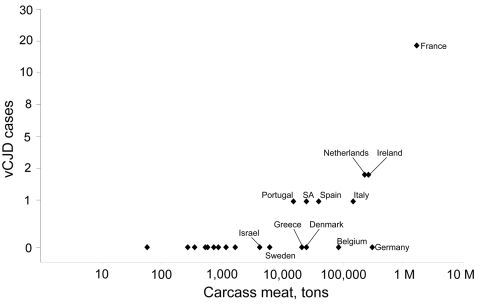
Scatter plot of the tonnage of carcass meat (unweighted data) imports from the United Kingdom (1980–1996) and the number of non-UK variant Creutzfeldt-Jakob disease (vCJD) cases per country. Values are logarithmic. M, million.

Figure 1 shows a scatter plot of the number of cases of indigenous BSE in non-UK countries and the number of non-UK vCJD cases per country. Although the confidence intervals (CIs) are wide, evidence of a correlation between these 2 variables (r_s_ 0.70, 95% CI 0.37–0.87, p = 0.001) exists in the countries belonging to the EU network (Table 2). When we included in our analysis Japan and Saudi Arabia, the only 2 countries outside the EU network in which vCJD cases have been detected, the correlation coefficient fell to 0.55 (95% CI 0.17–0.79, p = 0.008) (Table 2). Non-UK vCJD cases in EU network countries were also correlated with the number of live bovine imports from the UK (Figure 2; r_s_ 0.73, 95% CI 0.42–0.89, p<0.001) and the amount of carcass meat imported from the UK (Figure 3; r_s_ 0.75, 95% CI 0.45–0.89, p<0.001). Including Japan and Saudi Arabia produced similar results (live bovines r_s_ 0.65, 95% CI 0.31–0.84; carcass meat r_s_ 0.73, 95% CI 0.45–0.88) (Table 2).

**Table 2:** Results of nonparametric correlation analyses between number of variant Creutzfeldt-Jakob disease (vCJD) cases and the 3 studied exposure sources*

Countries and vCJD case-patients included in analysis	Exposure		
	Indigenous BSE cases (CI, p value)	Live bovines imported from UK, 1980–1990 (CI, p value)†	Carcass meat imported from UK, 1980–1996 (CI, p value)†
All EU network countries			
Patients likely to have been infected in UK excluded	r_s_ = 0.70 (CI 0.37–0.87, p = 0.001)	r_s_ = 0.73 (0.42–0.89, p<0.001)	r_s_ = 0.75 (0.45–0.89, p<0.001)
Patients likely to have been infected in UK included	r_s_ = 0.60 (0.21–0.82, p = 0.005)	r_s_ = 0.63 (0.26–0.84, p = 0.003)	r_s_ = 0.64 (0.27–0.84, p = 0.003)
All EU network countries plus Japan and Saudi Arabia			
Patients likely to have been infected in UK excluded	r_s_ = 0.55 (0.17–0.79, p = 0.008)	r_s_ = 0.65 (0.31–0.84, p = 0.001)	r_s_ = 0.73 (0.45–0.88, p<0.001)
Patients likely to have been infected in UK included	r_s_ = 0.51 (0.11–0.77, p = 0.02)	r_s_ = 0.52 (0.13–0.77, p = 0.01)	r_s_ = 0.57 (0.19–0.80, p = 0.006)
All countries except France			
Patients likely to have been infected in UK excluded	r_s_ = 0.48 (0.06–0.75, p = 0.03)	r_s_ = 0.60 (0.23–0.81, p = 0.005)	r_s_ = 0.68 (0.36–0.86, p = 0.001)
Patients likely to have been infected in UK included	r_s_ = 0.44 (0.01–0.73, p = 0.05)	r_s_ = 0.44 (0.01–0.73, p = 0.05)	r_s_ = 0.49 (0.08–0.76, p = 0.02)

*BSE, bovine spongiform encephalopathy; UK, United Kingdom; EU, European Union; CI, confidence interval; r_s_ Spearman rank correlation coefficient. †Weighted by the temporal distribution of the export in relation to the size of the BSE epizootic in the UK.

We evaluated whether our findings were dependent on the data from France, which has the largest number of non-UK cases, by repeating the analyses excluding France. Evidence remained that all 3 exposures were correlated with vCJD incidence (Table 2). We also repeated the analysis including the 6 cases detected outside the UK but thought to have been acquired in the UK. The inclusion of these cases resulted in a reduction in all 3 correlation coefficients (Table 2).

## Discussion

Live bovine and carcass meat imports from the United Kingdom during the 1980s and the first half of the 1990s correlate with the numbers of vCJD cases in countries outside the UK. This finding suggests that live bovine and/or carcass meat imports from the UK may have been an important source of exposure in at least some of the countries in which vCJD has been detected. These results are consistent with an analysis of data from France, which suggested that UK bovine imports were likely to have been a more important source of infection than indigenous BSE (*6*). Thus, a proportion of cases observed to date outside the UK may have been acquired through imports from the UK rather than by the patients’ exposure to indigenous BSE. The inclusion in the analysis of the 6 non-UK case-patients thought to be infected in the UK reduced all 3 correlation coefficients, as one would expect if the supposition that they were infected in the UK is correct.

These findings come with several important caveats. First, they are based on small numbers of vCJD cases. Even a small number of additional non-UK cases in the future could alter the findings substantially. Furthermore, we have not performed multivariable analyses to determine which of the 3 exposures of interest were correlated with vCJD incidence because we were concerned that the small number of cases might lead to unreliable results. Second, the analyses of imports are based on UK Customs and Excise data, not all of which been validated by importing countries. Even if these data are reasonably accurate, the actual level of BSE infection entering the human food chain in importing countries cannot be estimated because many important unknown variables exist, such as the age distribution of imported live bovines, the age at slaughter of these animals, the culinary habits in each country, and the possibility that some of the UK imports may have been re-exported to other countries. Third, indigenous BSE-infected cattle entering the food supply will have gone undetected until the introduction throughout the EU of the active abattoir testing program for BSE in 2000/2001; even now, cattle in the early stages of infection are unlikely to be detected. Furthermore, the efficiency of BSE surveillance varies from country to country: underascertainment is likely in those countries with limited or no active testing programs.

It is noteworthy that none of the 162 UK patients with vCJD identified up to September 2006 were born after 1989, the year in which the specified bovine offal ban was introduced to minimize human exposure to BSE; however, 2 of the 34 non-UK vCJD case-patients were born after 1989. Measures equivalent to the UK ban on specified bovine offals were not introduced in many continental European countries until 2000.

Despite these caveats, our results suggest that, globally, imports from the UK may have been an important source of infection and for some countries may even have been the main source. If this is so, our findings have several implications. Past UK exports may be the major determinant of the current incidence of vCJD outside the UK. The greatest volume of these exports was to France, the Netherlands, and Ireland. Thus, initially at least, we might expect the largest number of vCJD cases to occur in these countries. However, exposure to BSE through imports from the UK ceased in 1996, and exposure to indigenous BSE is likely to have continued at some level until the measures introduced in 2000. Thus, the proportion of vCJD cases due to exposure to indigenous BSE may increase with time.
